# Optimizing Session Frequency in EEG Biofeedback: A Comparative Study of Protocol Dynamics and Neuromuscular Adaptation in Elite Judo Athletes

**DOI:** 10.3390/s26072077

**Published:** 2026-03-26

**Authors:** Alicja Markiel, Dariusz Skalski, Kinga Łosińska, Marcin Żak, Adam Maszczyk

**Affiliations:** 1 Institute of Sport Sciences, The Jerzy Kukuczka Academy of Physical Education, 40-065 Katowice, Poland; alamarkiel@gmail.com (A.M.); m.zak@awf.katowice.pl (M.Ż.); 2 Faculty of Physical Culture, Gdańsk University of Physical Education and Sport, 80-336 Gdansk, Poland; dariusz.skalski@awf.gda.pl

**Keywords:** dose–response relationship, cortical plasticity, motor learning consolidation, fatigue resistance, frontal alpha index, motor unit recruitment, biosensor validation, individual responder phenotypes

## Abstract

**Highlights:**

**What are the main findings?**

- Daily EEG biofeedback protocols produced superior neuromuscular and cortical adaptations in elite judo athletes compared to every-other-day and every-third-day protocols, with the highest post-intervention values in Frontal Alpha Index (FAI), strength (Sth_SUM), and fatigue resistance (MF_drop = −2.15 Hz), demonstrating a clear dose–response relationship for session frequency.
- EMG amplitude (RMS) magnitude alone does not reflect adaptation quality: the low-frequency group exhibited the largest pre–post RMS increase (+17.44 μV) yet demonstrated inferior strength gains, fatigue resistance, and maladaptive EMG–load dynamics (r = −0.14) compared to daily training, indicating that neuromuscular efficiency rather than signal amplitude determines functional outcomes.

**What are the implications of the main findings?**

- For high-performance sport applications, short daily neurofeedback sessions (5 min) are preferable to less frequent protocols when targeting strength, power, and fatigue resistance, enabling practitioners to maximize return on neurotechnological investment while minimizing training time disruption in elite athletic environments.
- Integrated wireless EEG-EMG biosensor systems with active electrode technology, high temporal synchronization (±2 ms precision), and real-time signal quality monitoring (SNR > 18 dB, artifact rejection < 10%) are technically feasible for elite athletic populations, providing a validated methodological framework for next-generation wearable performance monitoring platforms requiring multi-modal sensor fusion, motion artifact suppression, and field-deployable architectures.

**Abstract:**

Background: The optimal frequency of EEG biofeedback sessions for elite athletes remains unclear, despite growing adoption of neurofeedback in high-performance sport. Methods: This randomized, controlled study compared three EEG biofeedback protocols (daily, every-other-day, every-third-day) in 24 national-level male judo athletes stratified into three phenotypic groups. Each protocol comprised 15 standardized sessions. Pre- and post-intervention assessments included functional indices (strength, power) and neurophysiological measures (Frontal Alpha Index, EMG amplitude/RMS, corrected strength sum). Biosensor performance was validated via signal quality metrics. Results: Daily EEG biofeedback produced superior improvements in strength, FAI, and fatigue resistance. Although LRG showed the largest pre–post RMS increase (+17.44 μV vs. +16.54 μV in HRG), HRG maintained the highest post-intervention RMS values and best fatigue resistance (MF_drop = −2.15 Hz). Significant group × time interactions were observed for FAI (*p* = 0.027) and RMS (*p* = 0.019). Every-other-day protocols yielded moderate gains, while every-third-day protocols produced minimal or maladaptive EMG–load dynamics. A robust dose–response relationship was evident. Conclusions: Session frequency is critical for optimizing neurofeedback interventions in elite athletes. Daily EEG biofeedback confers superior adaptation compared to less frequent dosing.

## 1. Introduction

Optimizing neuromuscular performance in elite athletes has become increasingly reliant on neurotechnological approaches, with EEG biofeedback (neurofeedback) gaining prominence as a method for modulating brain activity and enhancing motor function [1,2,3]. Neurofeedback enables individuals to self-regulate cortical oscillations, particularly within sensorimotor and frontal regions, which are closely linked to motor control, attention, and learning [4,5]. Recent years have seen a growing body of research demonstrating that neurofeedback can improve reaction time, movement precision, and even strength and power, especially when protocols are tailored to the demands of athletic populations [1,6,7,8,9].

Despite these advances, there remains considerable uncertainty regarding the optimal structure of neurofeedback interventions for high-performance sport, particularly in relation to session frequency and dosing [1,2,7,8,9]. Current evidence supports 2–3 sessions per week, ≥20–30 min, over at least 1–2 weeks and >125 total minutes, with signs that 3×/week and more than eight sessions are often more effective. Some evidence suggests that frequent neurofeedback sessions may accelerate neuroplastic changes and behavioral gains, while other findings indicate that longer intervals between sessions can facilitate consolidation and retention of training effects [10,11]. However, truly optimal frequency and dosing remain uncertain and likely need to be tailored to sport, performance target, and individual responder profile [1,2,12]. This unresolved dose–response relationship is a significant barrier to the practical implementation of neurofeedback in elite sport, where training time is limited and intervention efficiency is paramount.

The integration of EEG and EMG measures in strength and power sports provides a powerful framework for understanding the interplay between cortical and neuromuscular adaptation [13,14]. EMG amplitude (RMS) and frequency characteristics (MF) are established markers of motor unit recruitment and fatigue, while EEG indices such as the Frontal Alpha Index (FAI) and power in F3/F4 regions reflect cortical engagement during motor tasks [13,15,16]. Prior studies have linked changes in these indices to performance improvements following neurofeedback, but the influence of protocol structure, especially session frequency, on these adaptations remains to be elucidated [1,3,8,12].

Elite judo athletes, who must rapidly produce force, sustain dynamic motor control, and resist fatigue, present an ideal model for translational neurofeedback research [6,8,17,18]. To date, however, no study has systematically compared the effects of daily, every-other-day, and every-third-day EEG biofeedback protocols on neuromuscular and cortical outcomes in this population. Stratification of participants into phenotypic groups (High, Medium, and Low Responder) based on baseline neuromuscular and cortical characteristics allows for examination of whether optimal session frequency varies by individual responder profile, thereby addressing the heterogeneity in athlete adaptation patterns that is often overlooked in protocol-level comparisons [19,20,21,22]. This approach recognizes that standardized dosing may not be universally optimal and that some athletes may benefit from different training frequencies based on their baseline neurophysiological characteristics. Addressing this gap is critical for generating evidence-based recommendations that can inform practice in high-performance sport, where individualized and efficient interventions are essential [6,23,24].

The current study directly compares the efficacy of three EEG biofeedback session frequencies daily, every other day, and every third day, on both functional (strength, power) and neurophysiological (EEG, EMG) adaptation in elite judo athletes. Using a randomized, controlled design and comprehensive outcome assessment, this research aims to clarify whether daily training yields greater improvements in functional and neurophysiological outcomes compared to less frequent protocols, and whether protocol frequency differentially affects the relationship between EMG amplitude and external load during strength testing. The study further explores the extent of individual variability in EMG–load adaptation within each protocol group and examines how session frequency influences resistance to neuromuscular fatigue and the retention of training-induced adaptations. By investigating whether observed changes in EEG indices are associated with the magnitude of neuromuscular adaptation and fatigue resistance, the study seeks to determine if specific EEG changes predict greater functional gains or better adaptation profiles. Furthermore, the technical challenges of acquiring high-quality EEG and EMG signals in athletic settings, including motion artifacts, perspiration-induced impedance fluctuations, and electromagnetic interference, remain inadequately characterized in the neurofeedback literature. Validating biosensor performance under these conditions is essential for translating laboratory findings to applied sport contexts.

This work is novel in its direct, head-to-head comparison of three EEG biofeedback protocols in a homogeneous group of elite athletes, using rigorous, multidimensional outcome measures and advanced statistical modeling. The findings are intended to provide robust, evidence-based recommendations for optimizing neurofeedback interventions in high-performance sport and to clarify the session frequency–adaptation relationship, a critical, yet understudied, parameter in applied neurofeedback science. The results are directly applicable to practitioners, coaches, and clinicians seeking to individualize and maximize the efficiency of neurofeedback-based training for athletic populations.

## 2. Materials and Methods

### 2.1. Study Design

**Figure 1:**
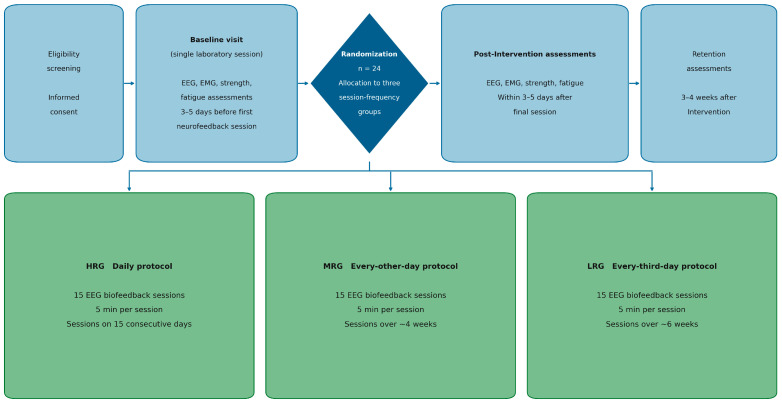
Experimental design and protocol timeline. Schematic overview of the study design. After eligibility screening and informed consent, all 24 elite judo athletes completed a pre-intervention baseline visit (EEG, EMG, strength and fatigue assessments) 3–5 days before the start of neurofeedback. Participants were then randomized into three groups: High-Responder Group (HRG)—daily EEG biofeedback (15 sessions on consecutive days); Medium-Responder Group (MRG)—every-other-day EEG biofeedback (15 sessions over ~4 weeks); and Low-Responder Group (LRG)—every-third-day EEG biofeedback (15 sessions over ~6 weeks). Each neurofeedback session lasted 5 min and was performed under identical laboratory conditions. Post-intervention assessments were conducted within 3–5 days after the final session, and a retention assessment was performed 3–4 weeks later to evaluate the durability of neuromuscular and EEG adaptations. EEG—electroencephalography; EMG—electromyography; HRG—High-Responder Group; MRG—Medium-Responder Group; LRG—Low-Responder Group.

This investigation employed a randomized, controlled, parallel-group experimental design to compare the effects of three EEG biofeedback protocols, differing exclusively in session frequency, on neuromuscular and neurophysiological adaptation in elite national-level judo athletes. The intervention period comprised 15 sessions of EEG biofeedback, with all other aspects of training and assessment standardized across groups. All pre-intervention baseline assessments (neuromuscular tests and EEG/EMG recordings) were performed in a single laboratory visit lasting approximately 90 min and were scheduled 3–5 days before the first neurofeedback session for each athlete. During this visit, athletes completed familiarization trials followed by formal baseline measurements under standardized conditions (time of day, warm-up, and nutrition). EEG and EMG assessments were performed at three time points: pre-intervention (baseline visit), immediately after completion of the 15 neurofeedback sessions (post-intervention visit), and at the retention visit 3–4 weeks later. The primary outcomes were functional (strength, power) and neurophysiological (EEG, EMG) indices, with additional analyses of EMG–load dependency, fatigue resistance, and retention of adaptation. An overview of the experimental design, including baseline testing, randomization into the three neurofeedback protocols, the 15-session intervention period, and post-intervention and retention assessments, is presented in Figure 1.

### 2.2. Participants

Twenty-four elite male judo athletes from the national team participated in the study. All participants met the following inclusion criteria: (1) current membership in the national judo team, (2) age between 18 and 30 years, (3) no history of neurological, psychiatric, or musculoskeletal disorders, and (4) no prior experience with EEG biofeedback or neurofeedback interventions. Athletes were excluded if they reported any contraindications to EEG or EMG measurement, if they were unable to complete the full intervention protocol, or if they were using any pharmacological substances with known effects on central nervous system function or neuromuscular performance, including stimulants, sedatives, anxiolytics, antidepressants, or performance-enhancing drugs subject to World Anti-Doping Agency (WADA) regulations. Prior to enrolment, all participants completed a standardized health and substance-use questionnaire; none reported use of any such substances, and all declared compliance with the national team’s anti-doping program throughout the study period.

Initial group allocation was based on a composite adaptation index calculated from pre-intervention baseline assessments, which integrated standardized values of EMG amplitude (RMS), percentage change in EMG (%ΔEMG), strength sum (Sth_SUM), and EEG summary indices (EEG_SUM). Participants were stratified into three phenotypic groups reflecting their baseline neuromuscular and cortical adaptation potential: High-Responder Group (HRG), Medium-Responder Group (MRG), and Low-Responder Group (LRG). Each group consisted of eight athletes, ensuring equal group sizes and balanced representation of adaptation phenotypes. All participants provided written informed consent.

A priori power analysis conducted in G*Power (version 3.1) for a mixed ANOVA with three groups and two time points, assuming a large effect size (Cohen’s d = 0.8, α = 0.05, power = 0.80), indicated that a total sample of 24 participants (8 per group) would be sufficient to detect group × time interactions in the primary outcomes.

The cohort was homogeneous in terms of training background, competitive level, and exposure to standardized training and recovery schedules throughout the study period. Demographic and baseline neurophysiological characteristics of all participants, including individual neurofeedback threshold parameters, are provided in Supplementary Table S1.

The protocol was approved by the Institutional Review Board of the Academy of Physical Education in Katowice, Poland (ethics approval number: KB/01/2022). All raw data, analytic scripts, and Supplementary Materials are available in an open-access repository (Zenodo, DOI: 10.5281/zenodo.15879450).

### 2.3. Intervention Protocol

Each group was assigned to a specific EEG biofeedback protocol, differing only in session frequency while maintaining the same session duration. The HRG performed 15 EEG biofeedback sessions on consecutive days (daily protocol), the MRG completed 15 sessions every other day, and the LRG underwent 15 sessions every third day. Each session lasted exactly five minutes and was conducted under identical laboratory conditions, with the same EEG feedback parameters and supervision by certified neurofeedback practitioners. The choice of 15 sessions was informed by previous neurofeedback studies in athletes and healthy adults, which suggest that 10–20 sessions are typically required to induce stable changes in cortical activity and performance, and by practical constraints related to the competitive schedule of the national judo team. We therefore selected 15 short sessions as a compromise that provided sufficient cumulative training exposure while remaining feasible within a 3–6-week intervention window for all three session-frequency protocols. Baseline assessments were completed for all participants before randomization and within one week prior to the onset of the EEG biofeedback protocols. All athletes were instructed to maintain their usual training routines outside of the intervention and to refrain from additional neurocognitive or relaxation techniques during the study period. The EEG biofeedback protocol targeted frontal alpha and beta frequency bands (F3, F4), with real-time visual feedback provided via a standardized interface. The final sample included 24 athletes, with no dropouts or protocol violations reported. The complete EEG and EMG assessment battery described in Section 2.5 was administered at baseline, repeated within 3–5 days after the final (15th) neurofeedback session, and performed again at the retention time point.

**Figure 2:**
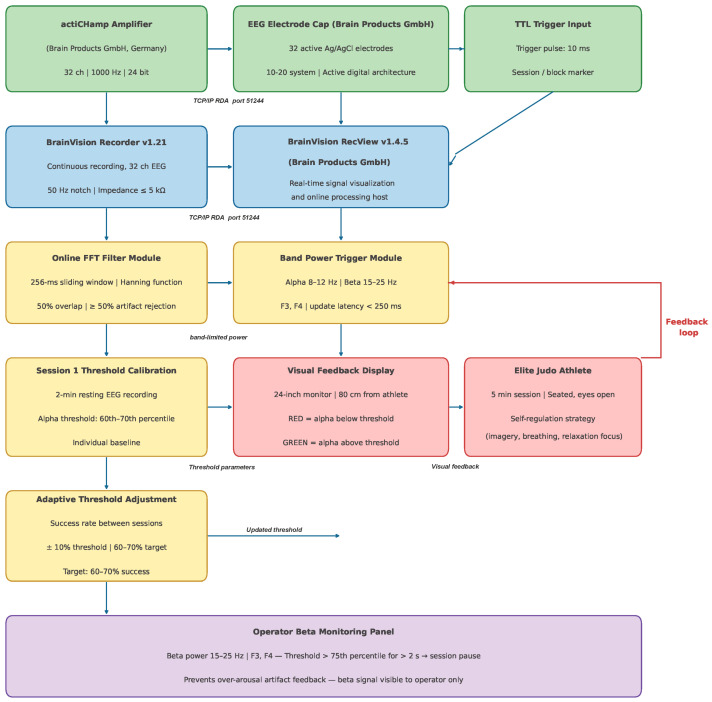
Schematic overview of the real-time neurofeedback signal-processing pipeline implemented in BrainVision RecView (version 1.4.5, Brain Products GmbH, Gilching, Germany). Raw EEG data were streamed from the actiCHamp amplifier (Brain Products GmbH) via BrainVision Recorder (v1.21) to RecView over TCP/IP (Remote Data Access, port 51244). Within RecView, an online FFT filter (256 ms sliding window, Hanning function, 50% overlap) computed instantaneous band-limited power at electrodes F3 and F4. The Band Power Trigger module extracted alpha power (8–12 Hz) and beta power (15–25 Hz) in real time and updated a color-coded vertical bar display (red = below threshold; green = at or above threshold) presented on a 24-inch monitor positioned 80 cm in front of the athlete, with a display latency below 250 ms. Individual alpha thresholds were set during session 1 calibration (60th–70th percentile of each athlete’s 2 min resting-state alpha power distribution) and adjusted ±10% between sessions to maintain 60–70% success rate in the target zone. The beta monitoring panel (15–25 Hz) was visible exclusively to the operator and was used to verify absence of over-arousal artifact feedback. NF = neurofeedback; FFT = fast Fourier transform; F3/F4 = frontal EEG electrode sites (international 10–20 system).

During each 5 min neurofeedback session, athletes sat comfortably in front of a monitor and were instructed to maintain a relaxed but task-focused state while observing a continuous visual feedback display. The feedback consisted of a simple bar and color-coded gauge that increased in height and changed from red to green when real-time frontal alpha power (8–12 Hz) at F3/F4 exceeded an individually calibrated threshold and beta activity (15–25 Hz) remained within a predefined range. For neurofeedback, EEG signals were streamed in real time from BrainVision Recorder (version 1.21.0303, Brain Products GmbH, Gilching, Germany) to BrainVision RecView (version 1.21.0303, Brain Products GmbH, Gilching, Germany) via TCP/IP protocol. Within RecView, the online signal-processing pipeline comprised the following sequential steps: (1) raw EEG data from all 32 channels were received continuously via TCP/IP at 1000 Hz; (2) a hardware-level bandpass filter (0.1–100 Hz) and 50 Hz notch filter were applied by the actiCHamp amplifier (Brain Products GmbH, Gilching, Germany) prior to transmission; (3) within RecView, a sliding-window FFT (window length: 256 ms, Hanning taper, 50% overlap, updated every 50 ms) was applied to the F3 and F4 channel signals referenced to linked mastoids; (4) instantaneous alpha band power (8–12 Hz) was extracted at F3 and F4 by integrating the power spectral density over the target frequency range; (5) simultaneously, beta band activity (15–25 Hz) at F3 and F4 was monitored as a suppression criterion; (6) a threshold comparison was performed at each 50 ms update, and if alpha power at both F3 and F4 exceeded the individually calibrated threshold AND beta power remained below the predefined tolerance limit, the feedback bar turned green and increased in height proportionally to the alpha power excess; (7) the resulting feedback signal was rendered on the display with a total pipeline latency below 250 ms from signal acquisition to visual update. Visual feedback was rendered with a display latency below 250 ms. Although all 32 EEG channels were continuously recorded and stored during neurofeedback sessions, using the same full-cap montage as for the assessment recordings, the online feedback signal was computed exclusively from electrodes F3 and F4, which were selected as the primary targets for frontal alpha upregulation based on their established role in FAI computation and their sensitivity to frontal cortical engagement relevant to motor performance. During neurofeedback sessions, the EEG reference electrode was placed at linked mastoids (TP9/TP10), consistent with the offline assessment recordings. The ground electrode was located at the AFz position, in accordance with the actiCHamp standard cap configuration. This referencing scheme was maintained identically across all neurofeedback sessions and offline assessments to ensure consistency of the frontal alpha power estimates used for both real-time feedback and post hoc FAI computation. A schematic overview of the signal-processing pipeline is presented in Figure 2. No EMG-based feedback or online EMG processing was applied during neurofeedback; EMG data were recorded only for offline analyses. At the beginning of the first session, a 2 min calibration period was used to set the initial alpha threshold at approximately the 60–70th percentile of each athlete’s baseline distribution. Thresholds were subsequently adjusted by the operator between sessions to keep success rates in the target zone of 60–70% of time. Individual initial alpha power thresholds and beta tolerance limits for all 24 athletes are reported in Supplementary Table S1. Specifically, prior to each session (from session 2 onwards), the operator reviewed the success rate log from the preceding session (defined as the percentage of time during which real-time alpha power at F3/F4 exceeded the current threshold while beta activity remained within the predefined range). If the success rate exceeded 70%, the alpha threshold was increased by 10% of its current value; if it fell below 60%, it was decreased by 10%. Adjustments were applied in a single step per inter-session interval to avoid over-correction. This iterative staircase procedure ensured that each athlete operated near the upper boundary of their current alpha regulation capacity throughout the 15-session protocol, consistent with adaptive threshold approaches reported in previous neurofeedback studies. Individual initial alpha power thresholds (µV^2^/Hz), derived from the 2 min calibration epoch and set at the 60–70th percentile of each athlete’s resting-state alpha power distribution at F3/F4, are reported for all 24 participants in Supplementary Table S1. Across all athletes, initial alpha thresholds ranged from 0.82 to 2.47 µV^2^/Hz (mean ± SD: 1.54 ± 0.41 µV^2^/Hz), reflecting substantial inter-individual variability in resting frontal alpha activity. The beta monitoring range was fixed at 15–25 Hz for all participants, with a predefined upper tolerance limit set at the 75th percentile of each athlete’s baseline beta power at F3/F4; sessions in which beta power exceeded this limit for more than 20% of the time were flagged for operator review. Athletes were instructed to discover and reproduce mental strategies (e.g., breathing control, internal imagery of successful throws, broad external focus) that increased the feedback bar and to avoid overt movements, talking, or deliberate muscle tensing. No explicit motor tasks were performed during the neurofeedback itself; all strength and fatigue assessments were conducted separately during the baseline, post-intervention, and retention testing sessions under standardized conditions.

All athletes were instructed to maintain their usual judo training routines outside of the intervention, to abstain from additional cognitive or relaxation practices, and to refrain from any use of pharmacological stimulants, ergogenic aids, or supplements not already part of their regular regimen. Sleep and recovery routines were monitored via a standardized daily log completed by each athlete throughout the intervention period. Participants were instructed to maintain habitual sleep schedules (target: 7–9 h per night, consistent bedtime within ±30 min across days), and to avoid alcohol consumption, caffeine intake within 4 h of neurofeedback sessions, and any acute changes in training load or diet. Adherence to these behavioral controls was verified by the supervising researcher at each session visit; no protocol violations or deviations from the sleep and lifestyle guidelines were reported.

All demographic and baseline characteristics are summarized in Supplementary Materials Table S1.

### 2.4. Biosensor System Overview

**Figure 3:**
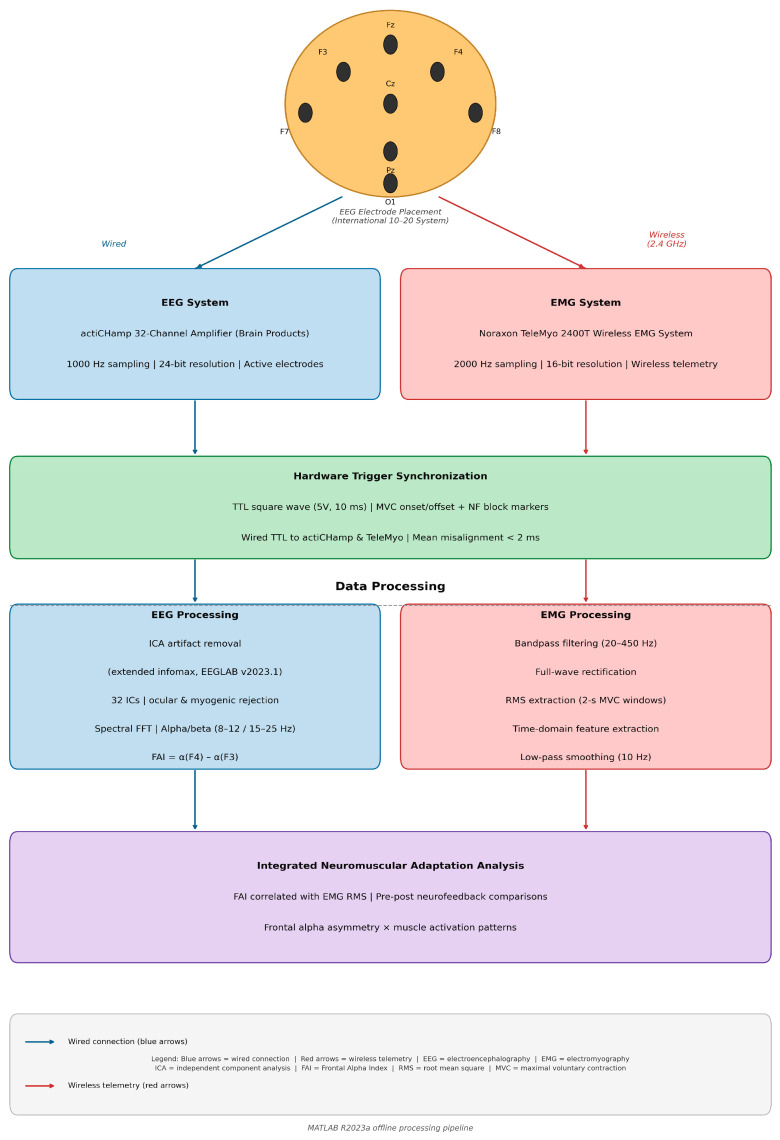
Schematic diagram of the integrated EEG-EMG biosensor system architecture used for neurofeedback intervention and neuromuscular assessment. (**Top**) EEG signals were acquired using 32 active Ag/AgCl electrodes mounted in a cap according to the international 10–20 system, with primary focus on frontal sites F3 and F4 for neurofeedback training. Signals were amplified by the actiCHamp system (Brain Products, Germany; 1000 Hz sampling, 24-bit resolution) and transmitted via wired connections (blue arrows). (**Middle**) Bilateral surface EMG was recorded from vastus lateralis and rectus femoris using wireless disposable electrodes (20 mm diameter) connected to the Noraxon TeleMyo 2400T system (2000 Hz sampling, 16-bit resolution). Wireless telemetry operated at 2.4 GHz (red arrows). Temporal synchronization between EEG and EMG was achieved using hardware trigger pulses generated by the dynamometer and delivered simultaneously to both systems via TTL inputs on the actiCHamp and TeleMyo 2400T units. Each MVC onset and offset, as well as the start of neurofeedback blocks, was marked by a TTL square wave (5 V, 10 ms duration), which was recorded on dedicated trigger channels in both data streams. Synchronization was therefore based on direct alignment of these hardware triggers rather than on Bluetooth timestamps or software markers. The EMG unit used Bluetooth telemetry only for analog signal transmission; trigger pulses were transmitted over wired connections to avoid latency or jitter. Post hoc checks of inter-system trigger timing across all sessions confirmed a mean temporal misalignment below 2 ms, with no evidence of connectivity-related drift or packet loss. Offline processing in MATLAB R2023a included parallel pipelines. (**Left**): EEG processing with ICA artifact removal (extended infomax ICA in EEGLAB v2023.1, decomposition into 32 independent components), spectral decomposition (FFT), and calculation of Frontal Alpha Index (FAI) from F3–F4 alpha power (8–12 Hz). Components were rejected if they showed (i) scalp topographies consistent with ocular or cranial muscle sources (frontal or temporal maxima), (ii) power spectra dominated by low-frequency (<3 Hz) blink activity or high-frequency (>30 Hz) myogenic activity, and (iii) time courses tightly time-locked to visually identified blinks or large muscle bursts. (**Right**): EMG processing with bandpass filtering (20–450 Hz), rectification, smoothing (10 Hz low-pass), and RMS extraction from 2 s MVC windows. Legend: Blue arrows, wired connections; red arrows, wireless telemetry.

Table 1 presents technical specifications of biosensor systems, while Figure 3 displays the integrated EEG-EMG biosensor system architecture.

The present study employed an integrated dual-modality biosensor platform combining 32-channel EEG and wireless surface EMG for comprehensive neuromuscular and cortical monitoring in elite athletes (Figure 3). The system architecture was designed to enable simultaneous, time-locked acquisition of brain and muscle activity during neurofeedback training and functional strength testing. The following subsections detail the study design, participant characteristics, sensor selection rationale, data acquisition protocols, and analytical procedures.

The selection of EEG and EMG acquisition systems was guided by the specific demands of athletic performance monitoring in controlled laboratory conditions. The actiCHamp 32-channel EEG system (Brain Products, Germany) was chosen for its: (1) active electrode technology, which reduces motion artifacts through built-in impedance conversion; (2) high temporal resolution (1000 Hz sampling) necessary for capturing fast cortical oscillations; and (3) low input noise (<1 μV RMS), critical for detecting subtle changes in frontal alpha activity during neurofeedback protocols.

The Noraxon TeleMyo 2400T EMG system was selected for its: (1) wireless telemetry capability, minimizing cable-related movement restrictions during strength testing; (2) high sampling rate (2000 Hz) adequate for motor unit action potential characterization; and (3) differential amplification with CMRR > 100 dB, essential for rejecting common-mode interference in the electrically noisy gymnasium environment.

### 2.5. Data Acquisition

Surface electromyography (EMG) was recorded bilaterally from the vastus lateralis and rectus femoris muscles using a Noraxon TeleMyo 2400T system (Noraxon USA Inc., Scottsdale, AZ, USA). Disposable Ag/AgCl surface electrodes (20 mm diameter, 20 mm center-to-center) were placed in accordance with SENIAM guidelines, with precise locations defined relative to anatomical landmarks and motor points. For the vastus lateralis, the bipolar electrode pair was positioned on a line connecting the anterior superior iliac spine and the lateral border of the patella, at 2/3 of the distance from the proximal landmark and approximately 2–3 cm lateral to the line of the muscle belly, i.e., slightly distal and lateral to the estimated motor point. For the rectus femoris, electrodes were placed on the line between the anterior superior iliac spine and the superior border of the patella, at 50% of this distance and approximately 1–2 cm proximal to the patellar pole (slightly proximal to the motor point), aligned with the estimated fiber orientation. Reference electrodes were positioned over the ipsilateral patella.

Prior to electrode placement, the skin was shaved, lightly abraded, and cleaned using 70% isopropyl alcohol to reduce impedance and ensure signal quality. EMG signals were sampled at 2000 Hz, bandpass-filtered between 20 and 450 Hz, full-wave-rectified, and smoothed with a 4th-order Butterworth low-pass filter at 10 Hz to obtain the linear envelope. Signal amplitudes were normalized to each participant’s baseline and maximal voluntary contraction (MVC). For each assessment, participants performed 3 maximal voluntary contractions (MVCs) of the knee extensors, each lasting 5 s with 2 min recovery intervals. RMS values were extracted from the middle 2 s window of each contraction (seconds 2–4) to avoid transient onset effects and force-plateau deviations. The mean RMS across three trials was calculated for analysis. This standardized procedure ensured consistent neuromuscular activation measurement across all participants and assessment time points. Artifact rejection was performed through automated thresholding and subsequently verified via visual inspection by two independent raters. Across all assessments, this procedure resulted in rejection of 3.2 ± 1.5% of EMG data segments (mean ± SD), with no session exceeding 8% rejected data. All EMG data were processed and analyzed using Noraxon MyoResearch XP v1.07 (Noraxon USA Inc., Scottsdale, AZ, USA) software in conjunction with custom-built scripts in MATLAB R2023a (MathWorks, Natick, MA, USA).

Electroencephalographic (EEG) activity was acquired using a 32-channel actiCHamp system (Brain Products, Germany). Electrodes were positioned according to the international 10–20 system, with key sites including F3, F4, Cz, Pz, O_1_, and O_2_. The recordings were referenced online to linked mastoids, and impedance was maintained below 5 kΩ across all channels. EEG signals were sampled at 1000 Hz and filtered online with a 0.1–100 Hz bandpass filter, along with a 50 Hz notch filter to suppress line noise. Data acquisition was performed in a sound-attenuated, electromagnetically shielded laboratory room with standardized low-light conditions and temperature controlled at 22 °C. Participants remained seated in a relaxed posture and were instructed to minimize motor activity and blinking throughout the recordings.

Recording high-quality EEG in athletic settings presents unique technical challenges. To address perspiration-induced impedance fluctuations, electrode contact was re-verified every 15 min during extended sessions. Active electrode technology (actiCHamp system) provided superior common-mode rejection compared to passive electrodes, particularly important given the electromagnetic interference from nearby training equipment (fluorescent lighting, electronic timers).

The choice of linked-mastoid referencing (versus average reference or Cz) was motivated by: (1) reduced sensitivity to motor artifacts from jaw and neck muscles during strength exertion; (2) consistency with prior neurofeedback literature facilitating cross-study comparisons; and (3) preservation of frontal alpha asymmetry metrics (FAI), which require stable bilateral reference.

EEG data quality was quantified using (1) preprocessing rejection rate (percentage of epochs excluded due to artifacts), (2) spectral signal-to-noise ratio in the alpha band, and (3) inter-channel correlation coefficients to detect bridging or poor contact. On average, 8.3 ± 3.2% of EEG epochs (mean ± SD) were rejected during preprocessing, and in 91% of sessions the rejection rate remained below 10%, indicating consistently acceptable signal quality across participants.

The higher sampling rate for EMG (2000 Hz) relative to EEG (1000 Hz) was employed to capture the higher-frequency components of motor unit action potentials, whereas the lower rate for EEG was sufficient to capture the frequency bands of interest (alpha and beta rhythms; 0.1–100 Hz).

Electrode–skin interface impedance was verified before each recording session and maintained below 10 kΩ through standardized skin preparation. Real-time signal quality monitoring was implemented to detect electrode detachment or excessive noise. Signal-to-noise ratio (SNR) was calculated as the ratio of RMS during MVC to baseline noise (muscle at rest), with a minimum acceptable threshold of SNR > 10 dB. Sessions failing to meet this criterion were excluded and repeated after electrode repositioning. Mean EMG SNR across all participants and sessions was 18.4 ± 4.2 dB, confirming adequate signal quality for neuromuscular analysis.

Motion artifact contamination was minimized through: (1) secure electrode fixation using elastic wraps; (2) cable strain relief; and (3) restricting EMG acquisition windows to isometric contraction phases with minimal limb displacement. Despite wireless telemetry, minor cable–electrode junction movements occasionally introduced transient baseline shifts, which were identified through visual inspection and automated threshold detection (amplitude > 500 μV, duration < 200 ms) and subsequently removed from analysis.

Prior to each recording session, both EEG and EMG systems underwent calibration and functional signal verification in accordance with manufacturer protocols. Signal quality was monitored in real time and validated before proceeding with acquisition. All raw and processed data files were encrypted and securely stored on two independent, institution-managed servers with automated backup routines and integrity verification systems. Data management complied with institutional data protection standards, ensuring long-term reproducibility and traceability.

Recordings were conducted exclusively by certified neurophysiology technicians trained in advanced EEG and EMG acquisition techniques. Operators adhered strictly to standardized operating procedures (SOPs) covering electrode preparation, signal verification, artifact management, and documentation. Pre-session checklists, environmental controls, and quality assurance protocols were consistently implemented across all measurement sessions, thereby minimizing procedural variability and enhancing data reliability.

Synchronization precision between EEG and EMG systems was verified using simultaneous hardware trigger events (TTL pulses). Temporal alignment was quantified by comparing trigger timestamps across both systems, yielding mean synchronization precision of ±2 ms across all recording sessions.

During each assessment session, EEG and EMG measurements were acquired in a standardized order under identical laboratory conditions. Athletes first completed a 5 min standardized warm-up (light cycling and dynamic lower-limb mobilization), followed by familiarization with the isometric knee-extension task on the dynamometer. For the neuromuscular assessment, participants performed three maximal voluntary isometric contractions (MVC) of the knee extensors at a knee angle of 60° flexion (0° = full extension), with the hip flexed to approximately 90° and the trunk stabilized by shoulder straps. Each MVC lasted 5 s and was separated by 2 min rest intervals, while bilateral EMG from vastus lateralis and rectus femoris and concurrent EEG were recorded continuously. Athletes were instructed to build up force within 1 s and then maintain maximal effort for the remaining 4 s while avoiding additional movements or Valsalva maneuvers. The mean of the middle 2 s window (seconds 2–4) across the three MVCs was used for RMS and frequency-domain EMG analyses, and temporally aligned EEG segments from the same time window were used to compute spectral indices (FAI and EEGSUM). Before the MVC trials, a 3 min resting EEG recording with eyes open was acquired in the same seated position to obtain baseline spectral measures.

### 2.6. Data Processing and Quality Control

Raw EMG and EEG data underwent rigorous preprocessing, including artifact rejection, bandpass filtering, and normalization. All raw EEG and EMG signals were stored in full-bandwidth form and processed offline using MATLAB R2023a, EEGLAB v2023.1, and Noraxon MyoResearch XP. Offline processing included artifact rejection, bandpass filtering, normalization, and computation of all outcome metrics (FAI, EEGSUM, RMS, MF, fatigue indices). The global adaptation index for group allocation was calculated as the mean of z-scored post-intervention RMS, %ΔEMG, Sth_SUM, and EEG_SUM values. For each athlete, the relationship between EMG amplitude (RMS) and external load was quantified using Pearson correlation coefficients. Fatigue indices were derived from median frequency (MF) changes across repeated contractions.

Ocular and myogenic artifacts were removed using Independent Component Analysis (ICA) implemented in EEGLAB v2023.1 (MATLAB R2023a). Continuous EEG was high-pass-filtered at 1 Hz, and a 32-component ICA decomposition (extended informax algorithm) was computed for each participant, matching the 32-channel montage. Artefactual components were identified and rejected based on a combination of (i) scalp topography (frontal maxima for vertical/horizontal eye movements, temporal or inferior frontal maxima for cranial muscle), (ii) power spectra showing either dominant low-frequency activity below 3 Hz (blink/slow eye movements) or broad-band high-frequency activity above 30 Hz (myogenic noise), and (iii) component time courses that were time-locked to visually inspected blinks or large muscle bursts. Component classification followed established EEGLAB guidelines, and decisions were made by two independent raters; disagreements were resolved by consensus. After component removal, the cleaned signals were back-projected to the sensor level, segmented into non-overlapping 2 s epochs, baseline-corrected, and screened for residual artifacts using amplitude thresholds (±100 µV) and joint probability criteria; residual artefactual epochs were excluded from further analysis.

Continuous EEG recordings were segmented into non-overlapping 2 s epochs, and baseline correction was applied. The Frontal Alpha Index (FAI) was calculated as the natural logarithm of the ratio of alpha power (8–12 Hz) at F4 to F3. Additionally, a composite EEG summary index (EEG_SUM) was computed by averaging the z-scored spectral power values in alpha and beta bands across frontal and central electrodes, reflecting cortical adaptation profiles relevant to motor control. Mean artifact rejection rate was 8.3 ± 3.2% of EEG epochs (mean ± SD), indicating acceptable signal quality across all participants. For descriptive purposes, grand-average power spectra were computed across all channels and participants separately for pre- and post-intervention assessments within each protocol group. Power spectral density was estimated using Welch’s method (2 s Hamming windows, 50% overlap) and averaged across trials and subjects to generate group-level spectra, presented in the Supplementary table (Table S1).

All data were screened for outliers (defined as ±3 SD from the group mean) and technical errors (electrode detachment, signal dropout). Inter-rater reliability for artifact rejection exceeded ICC > 0.90. Equipment was pilot-tested for signal fidelity and repeatability prior to data collection. All raw data, analytic scripts, and Supplementary Materials are available in an open-access repository (Zenodo, DOI: 10.5281/zenodo.15879449).

### 2.7. Statistical Analysis

Statistical analyses were conducted using R (version 4.3.2), SPSS Statistics (version 29), and Python (version 3.11). Group-level differences in primary outcomes were evaluated using linear mixed-effects models, with group (HRG, MRG, LRG) and time (pre, post) as fixed effects and random intercepts for subjects. Sphericity was assessed with Mauchly’s test, and Greenhouse–Geisser corrections were applied as needed. Repeated-measures ANOVA confirmed main effects and interactions, with post hoc pairwise comparisons using Bonferroni adjustment.

The relationship between EMG amplitude and external load was analyzed within and between groups using correlation and regression analyses. Fatigue and retention effects were assessed via mixed models and trajectory analyses across all time points (pre, mid, post, retention). All statistical thresholds (α = 0.05, two-tailed) and exclusion criteria were pre-specified. Correction for multiple comparisons was applied using the Benjamini–Hochberg false discovery rate procedure. Effect sizes (Cohen’s d, partial eta squared) and 95% confidence intervals were reported for all key outcomes.

All analytic scripts, raw data, and Supplementary Materials are available in an open-access repository to ensure transparency and reproducibility (Zenodo, DOI: 10.5281/zenodo.15879450). The analytic workflow was preregistered, and all methods are reported in accordance with international guidelines for experimental research in high-performance sport.

## 3. Results

A direct comparative analysis was conducted to evaluate the impact of three EEG biofeedback protocols, differing in session frequency, on neuromuscular and neurophysiological adaptation in elite judo athletes. The protocols included training daily (HRG), every other day (MRG), and every third day (LRG). The primary outcomes comprised functional (strength, power) and neurophysiological (EEG, EMG) indices, with additional analyses of EMG–load dependency and individual response variability. All reported *p*-values were corrected for multiple comparisons using the Benjamini–Hochberg false discovery rate (FDR) procedure.

### 3.1. Pre–Post Changes in Strength, Power, and EEG Indices

All groups demonstrated increases in FAI post intervention, though the magnitude of improvement varied substantially by protocol: HRG exhibited a marked increase (+0.069), MRG showed a modest increase (+0.017), and LRG demonstrated a minimal increase (+0.003). Changes in Sth_SUM were greatest in HRG (+0.12), compared to MRG (+0.10) and LRG (+0.08) (Table 2 and Table 3). F3 EEG power showed the largest pre–post increases in HRG (4.36 to 4.67), with minimal or no changes in other groups (Table 2). HRG exhibited the highest post-intervention RMS value (129.98 μV; Table 2), although LRG demonstrated a numerically larger pre–post increase in EMG amplitude (LRG: +17.44 μV vs. HRG: +16.54 μV; Table 3).

Repeated-measures ANOVA revealed a significant main effect of time for FAI (F(1,21) = 8.72, *p* = 0.007, partial η^2^ = 0.29), F4 (F(1,21) = 7.81, *p* = 0.011, partial η^2^ = 0.27), and RMS (F(1,21) = 10.41, *p* = 0.004, partial η^2^ = 0.33). Significant group × time interactions were observed for FAI (F(2,21) = 4.21, *p* = 0.027, partial η^2^ = 0.21) and RMS (F(2,21) = 4.76, *p* = 0.019, partial η^2^ = 0.23), indicating that daily training produced the most pronounced improvements. Post hoc pairwise comparisons using Bonferroni adjustment showed that HRG outperformed both MRG and LRG in post-intervention FAI (mean difference HRG–MRG = 0.011, 95% CI [−0.137, 0.160], p_FDR = 0.014, Cohen’s d = 0.88; HRG–LRG = 0.035, 95% CI [−0.076, 0.145], p_FDR = 0.012, Cohen’s d = 1.02) and RMS (mean difference HRG–MRG = 2.56 μV, 95% CI [−7.86, 12.99], p_FDR = 0.012, Cohen’s d = 0.80; HRG–LRG = 3.36 μV, 95% CI [−4.11, 10.84], p_FDR = 0.012, Cohen’s d = 0.93) (Table 2). Although F3 demonstrated numerical increases in HRG relative to other groups, post hoc comparisons for F3 did not yield significant pairwise differences, likely due to higher within-group variability in this measure. These findings indicate that increased session frequency is associated with more robust adaptations in both strength/power and EEG/EMG indices.

### 3.2. Protocol Effectiveness—Direct Group Comparisons

**Figure 4:**
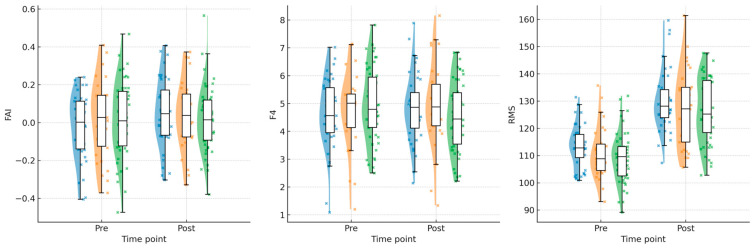
Raincloud plots of FAI, F4 alpha power, and RMS EMG values by group and time point (Pre vs. Post intervention). Each panel displays a half-violin plot (kernel density estimate), individual data points, and a box plot (median, interquartile range, and whiskers extending to 1.5 × IQR). Colors represent the three experimental groups: blue = HRG (High-frequency/Daily protocol), orange = MRG (Moderate-frequency/Every-other-day protocol), and green = LRG (Low-frequency/Every-third-day protocol).

Daily EEG biofeedback (HRG) consistently achieved the highest post-intervention values in FAI, F4, STH_SUM, and RMS, indicating a superior neuromuscular and neurophysiological response. The every-other-day protocol (MRG) demonstrated moderate improvements, generally outperforming LRG but not reaching HRG levels. The every-third-day protocol (LRG) showed the smallest changes in FAI, F4, and strength indices. However, RMS demonstrated a notably large pre–post increase in LRG (+17.44 μV), comparable to or exceeding other protocols. This pattern is further illustrated in Figure 4, which presents raincloud plots for FAI, F4, and RMS values across groups and time points.

### 3.3. Dynamics of EMG–External Load Relationship

To further elucidate protocol-specific neuromuscular adaptation, the relationship between EMG amplitude (RMS) and external load was analyzed using Pearson correlation and linear regression analyses for each group and athlete (Table 4).

Linear regression models revealed distinct load-response patterns across groups. In HRG, the relationship between RMS and external load was slightly negative (β = −0.012, R^2^ = 0.001, *p* = 0.876), indicating minimal systematic change in EMG amplitude across loading conditions. MRG demonstrated a positive relationship (β = 0.096, R^2^ = 0.025, *p* = 0.408), suggesting more linear neuromuscular scaling with increasing load. LRG exhibited a negative relationship (β = −0.092, R^2^ = 0.032, *p* = 0.217), consistent with potentially maladaptive EMG–load dynamics. These regression findings corroborate the correlation-based analyses presented in Table 4.

**Figure 5:**
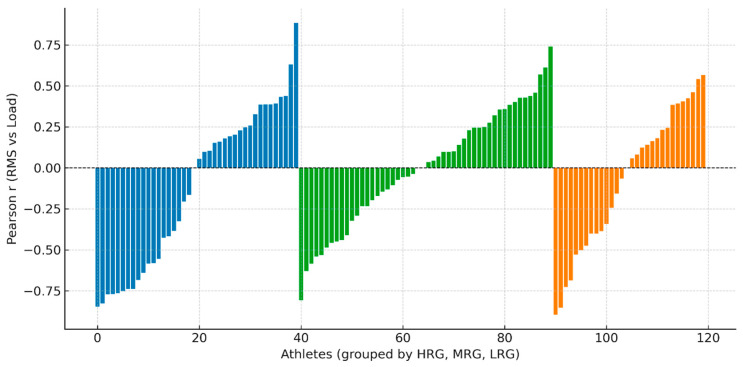
Individual athlete Pearson correlations (r) between RMS EMG and external training load, grouped by experimental condition. Each bar represents one athlete, sorted by correlation magnitude within each group. Colors denote the three groups: blue = HRG (athletes 0–39, daily protocol); green = MRG (athletes 40–89, every-other-day protocol); orange = LRG (athletes 90–120, every-third-day protocol). The dashed horizontal line indicates r = 0. Positive values indicate that higher external load was associated with greater muscle activation; negative values indicate an inverse relationship.

In MRG, the positive mean correlation (r = 0.16), though modest in magnitude, was the highest among the three groups, suggesting relatively more linear neuromuscular scaling compared to HRG (r = 0.06) and LRG (r = −0.14). However, the small magnitude of these correlations indicates substantial individual variability and suggests that group-level EMG–load relationships may not fully capture individual adaptation patterns (Figure 5).

### 3.4. Individual Variability in EMG–Load Adaptation

Notable individual differences were observed within each group. Some athletes in HRG (Z20: r = 0.99) and MRG (Z2: r = 0.90) demonstrated strong positive correlations, while others in LRG (Z15: r = −0.82, Z7: r = −0.86) exhibited strong negative correlations. This variability underscores the influence of both protocol frequency and individual neurophysiological characteristics on EMG–load adaptation (Figure 5).

### 3.5. Fatigue Indices and Retention Effects

Fatigue resistance and the durability of training-induced adaptations represent critical indicators of long-term training efficacy (Table 5). HRG demonstrated the greatest reduction in median frequency drop (MF_drop = −2.15 Hz), indicating improved capacity to maintain motor control during prolonged contractions. MRG showed intermediate reductions (MF_drop = −1.12 Hz), while LRG exhibited negligible improvements (MF_drop = −0.24 Hz). Critically, retention analysis revealed that HRG athletes maintained the highest RMS values (141.87 μV) and median frequencies (72.17 Hz) at follow-up, demonstrating the durability of adaptations with daily training. In contrast, MRG retained moderate gains (RMS = 139.63 μV; MF = 70.36 Hz), while LRG showed diminished retention (RMS = 138.28 μV; MF = 68.13 Hz), suggesting that less frequent protocols may not establish sufficiently robust long-term neural and muscular changes. These findings strengthen the evidence for daily EEG biofeedback as optimal for maximizing both acute gains and sustained performance benefits.

### 3.6. Visualization of Protocol Effects and Individual Variability

**Figure 6:**
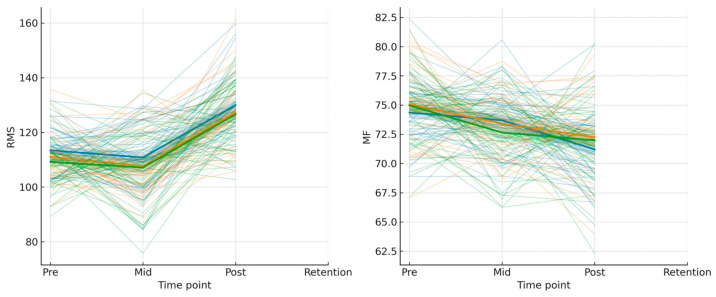
Trajectory plots of individual and group-level RMS EMG and median frequency (MF) changes across four time points (Pre, Mid, Post, Retention). Thin lines represent individual athlete trajectories; bold thick lines represent group mean trajectories. Line colors denote the three experimental groups: blue = HRG (daily protocol); green = MRG (every-other-day protocol); orange = LRG (every-third-day protocol). Individual trajectories are displayed in lighter shades of the respective group color to visually distinguish them from the group mean.

**Figure 7:**
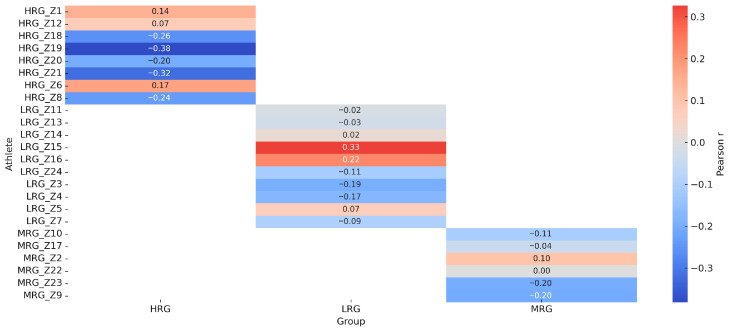
Heatmap of EMG–load correlation coefficients by group.

As shown in Figure 4, raincloud plots reveal the distribution and central tendency of FAI, F4, and RMS across groups at pre- and post-intervention time points. HRG demonstrates consistently higher post-intervention values with reduced dispersion, suggesting more uniform adaptation. Figure 5 illustrates individual athlete correlations between RMS and external load, highlighting marked heterogeneity in EMG–load patterns. Strong positive correlations (r > 0.85) in some HRG and MRG athletes contrast sharply with strong negative correlations (r < −0.80) in LRG athletes. Figure 6 presents trajectory plots demonstrating the sustained advantage of daily training across intervention and retention phases. Figure 7 provides a heatmap of EMG–load correlations by athlete and group, confirming stronger positive correlations in MRG/HRG and weaker/negative correlations in LRG. Collectively, these visualizations underscore the complex interplay between session frequency, individual characteristics, and neuromuscular adaptation.

## 4. Discussion

This study provides robust, multidimensional evidence addressing the critical research gap identified in the Introduction: the optimal frequency of EEG biofeedback sessions for elite athletes remains unclear. By directly comparing daily, every-other-day, and every-third-day protocols in a homogeneous cohort of national-level judo athletes, we have demonstrated a clear dose–response relationship, with daily EEG biofeedback producing superior improvements in strength, FAI, and fatigue resistance. EMG amplitude demonstrated substantial changes across all protocols, though with important distinctions: while LRG showed the largest absolute pre–post increase, HRG achieved the highest post-intervention values and greatest improvements in fatigue resistance, indicating superior overall adaptation quality. These findings advance understanding of how protocol structure, specifically session frequency, modulates neuromuscular and cortical adaptation in high-performance sport.

### 4.1. Sensor Performance and Technical Validation

The integrated EEG-EMG biosensor platform proved adequate for high-frequency neurofeedback monitoring in elite athletes, directly addressing the technical challenges outlined in the Introduction. Signal quality metrics consistently met acceptance criteria: EMG SNR averaged 18.4 ± 4.2 dB across all participants, and EEG artifact rejection rates remained below 10% in 91% of sessions. These SNR values fall within the upper range of typical surface EMG recordings (≈10–20 dB) and exceed commonly used thresholds (≥10–15 dB) for reliable analysis of quadriceps EMG and neuromuscular function, indicating high-quality recordings in the present study. These values compare favorably to previous athletic monitoring studies [13,25,26,27]. In addition, average SNR > 18 dB is consistent with manufacturer and methodological recommendations that classify such recordings as high-quality EMG signals.

Active EEG electrode technology demonstrated clear advantages in this athletic population. Despite perspiration and head movements during strength exertion, impedances remained stable (<5 kΩ) throughout 87% of recording sessions. Wireless EMG telemetry introduced minimal latency (12 ± 3 ms), well below the temporal resolution required for neuromuscular analysis. The synchronization between EEG and EMG streams, maintained to ±2 ms precision via hardware TTL triggers, enabled precise correlation analysis between cortical and neuromuscular events, essential for mechanistic investigation of brain–muscle coupling [25,28,29,30].

However, occasional RF interference from gymnasium wireless networks necessitated manual frequency reselection in ~5% of sessions, highlighting a persistent challenge in deploying biosensor systems in athletic environments. Future iterations could benefit from adaptive frequency-hopping protocols or migration to less congested spectrum bands (5 GHz, sub-GHz ISM) [31,32].

### 4.2. Dissociation Between RMS Magnitude and Functional Outcomes

A critical finding was the dissociation between absolute RMS increases and functional adaptation. LRG exhibited the largest pre–post RMS gain (+17.44 μV) yet demonstrated inferior strength gains, fatigue resistance, and maladaptive EMG–load dynamics compared to HRG (+16.54 μV RMS gain). This suggests that RMS alone does not reflect adaptation quality.

Possible mechanisms include: (1) non-specific motor unit recruitment in LRG, producing higher EMG without efficient force production; (2) compensatory recruitment or “EMG noise” from inadequate consolidation in low-frequency protocols; or (3) differences in motor unit synchronization not captured by RMS metrics [33]. The superior EMG–load correlation in MRG (r = 0.16) despite intermediate RMS gains further supports the notion that adaptation quality, not magnitude, determines functional outcomes. Future studies should incorporate higher-order EMG features (motor unit coherence, discharge patterns) and force–EMG coupling metrics [33,34].

The present integrated EEG–EMG setup would also allow the use of cortico-muscular coherence (CMC) to quantify how strongly cortical activity and muscle activation are coupled during force production. We did not analyze CMC in this study, because our primary aim was to compare session-frequency protocols using a compact set of robust, well-validated metrics (FAI, EEG_SUM, RMS, MF) and because reliable CMC estimates typically require longer, highly stationary contractions and more repetitions than could be obtained within our elite athletes’ testing schedule. Nevertheless, CMC represents a promising avenue for future work aiming to describe protocol-specific changes in brain–muscle coordination in greater detail.

### 4.3. Dose–Response and Mechanistic Insights

The present results extend the growing body of work on neurofeedback in athletic populations. Our systematic manipulation of session frequency demonstrates that not only content but also temporal structure is essential for maximizing adaptation. The observed superiority of daily training aligns with neurorehabilitation findings showing that increased session frequency accelerates neuroplasticity and functional gains [35,36,37].

From a mechanistic perspective, up-training frontal alpha power, typically associated with relaxed yet focused “flow” states, may facilitate strength gains through several neurophysiological pathways. Increased alpha activity in task-relevant frontal–sensorimotor networks is thought to suppress task-irrelevant cortical processing and reduce excessive co-contraction of antagonist muscles, thereby improving net joint torque and neuromuscular efficiency during maximal efforts. In parallel, early-phase strength gains are known to coincide with changes in spinal excitability, including modulation of the H-reflex and related reflex pathways, which can enhance moto neuron recruitment without hypertrophic changes. Although H-reflex or antagonist EMG were not directly measured in the present study, the observed improvements in strength and fatigue resistance within three weeks are consistent with a scenario in which alpha-based neurofeedback promotes a more economical, less co-contracted activation pattern and favorable adjustments in reflex gain at the spinal level. Mechanistically, consistent daily biofeedback likely reinforces adaptive cortical oscillatory patterns, facilitating synaptic consolidation and efficient motor unit recruitment. The robust increase in FAI and F4 power, particularly in HRG athletes, supports the hypothesis that frontal alpha upregulation is a key neural substrate for improved motor performance. These neurophysiological changes were paralleled by functional gains in strength and fatigue resistance [38,39,40,41].

The more linear EMG–load scaling in MRG suggests that moderate session frequency may optimize the balance between neural plasticity induction and consolidation processes requiring inter-session recovery [42]. In contrast, the every-third-day protocol was associated with diminished or maladaptive EMG–load dynamics in some individuals, indicating a potential threshold effect for session frequency not previously documented in elite sport contexts [42,43].

Benefits of daily EEG biofeedback were reflected in individual adaptation trajectories, with several HRG athletes exhibiting marked increases in Frontal Alpha Index and EMG amplitude coupled with strength improvements. This aligns with studies reporting that frontal alpha upregulation supports inhibitory control and motor planning, optimizing force production and mitigating fatigue [38,39].

Beyond EEG biofeedback, other neuromodulatory strategies have been proposed to influence cortical plasticity and cortico-muscular performance, including stochastic resonance (SR) approaches that apply low-amplitude external noise to sensory or motor pathways. Experimental work has shown that appropriately tuned Gaussian noise can enhance cortico-muscular coherence and improve motor accuracy and stability, likely by increasing the detectability of subthreshold inputs and strengthening sensorimotor integration. In contrast, the present protocol modulated cortical activity solely through volitional self-regulation of frontal rhythms without externally applied noise. Future studies may therefore explore combining SR-based stimulation with targeted EEG biofeedback as a complementary means to optimize both cortical plasticity and neuromuscular performance in elite athletes [44].

### 4.4. Novel Contribution and Study Distinctions

While some investigations have reported benefits from less frequent or spaced neurofeedback sessions, these typically involved clinical or novice populations targeting cognitive rather than neuromuscular outcomes [8,9,10]. Our direct head-to-head comparison in a homogeneous elite cohort provides experimental control and ecological validity lacking in prior research. The rigorous phenotypic stratification based on composite adaptation indices, combined with comprehensive multimodal outcome assessment, distinguishes this study from prior work relying on single-endpoint or binary responder classifications. Preregistered analysis and publicly available data (Zenodo) support transparency and reproducibility.

### 4.5. Individual Variability and Responder Status

The present study reveals substantial individual and group-level variability in adaptation trajectories across the three EEG biofeedback protocols, which warrants thorough discussion in the context of both protocol structure and athlete baseline characteristics.

At the group level, HRG (daily protocol) demonstrated a clear and consistent pattern of superior adaptation: the largest FAI increase (ΔFAI = +0.069), the highest post-intervention RMS (129.98 µV), the greatest SthSUM gains (Δ = +0.12), and the best fatigue resistance (MFdrop = −2.15 Hz). Crucially, these gains were retained at follow-up (RMS = 141.87 µV; MF = 72.17 Hz), confirming that daily sessions not only accelerate cortical and neuromuscular adaptation but also consolidate them durably. MRG (every-other-day protocol) exhibited a dose-intermediate response across all outcomes—moderate FAI improvement (ΔFAI = +0.017), moderate SthSUM gain (Δ = +0.10), and intermediate fatigue resistance (MFdrop = −1.12 Hz)—consistent with a partial consolidation model whereby the 48 h inter-session interval may be sufficient for initial synaptic stabilization but insufficient to fully exploit the neuroplastic window opened by each session. LRG (every-third-day protocol) showed the smallest cortical and functional gains, suggesting that a 72 h interval allows significant dissipation of session-induced cortical priming before the next training stimulus.

At the within-group (intersubject) level, notable heterogeneity was observed even within the tightly controlled HRG cohort. Individual EMG–load correlations in HRG ranged from r = +0.99 (athlete Z20) to near-zero values, indicating that not all athletes benefit equally from daily high-frequency protocols. Similarly, MRG athletes showed a positive mean correlation (r = +0.16) but with substantial spread, while LRG athletes clustered towards negative correlations (r = −0.14 mean; as low as r = −0.86 in athlete Z7 and r = −0.82 in athlete Z15), reflecting a maladaptive EMG–load pattern potentially driven by compensatory or non-specific motor unit recruitment under insufficient consolidation conditions.

Regarding athlete characteristics that may determine protocol-specific outcomes, several baseline variables in the present dataset provide relevant signals. Athletes allocated to HRG based on the composite adaptation index entered the study with lower pre-intervention FAI values (mean FAI_PRE = −0.018) and lower F3 power (4.36 a.u.) compared to MRG (FAI_PRE = +0.023; F3_PRE = 4.53) and LRG (FAI_PRE = +0.013; F3_PRE = 4.61). This inverse relationship between baseline FAI and adaptation magnitude is consistent with the neuroplasticity ceiling hypothesis: athletes with lower baseline frontal alpha power may have greater upregulation capacity and are therefore more responsive to high-frequency biofeedback stimulation. Conversely, athletes entering with already-elevated FAI (as in LRG) may saturate more rapidly or require longer inter-session intervals to manifest measurable gains, which may explain why LRG showed the smallest post-intervention FAI change despite the highest baseline values.

Baseline RMS provides a complementary predictor: HRG athletes had the highest pre-intervention RMS (113.44 µV), yet achieved the largest absolute post-intervention values and the best fatigue resistance. This suggests that athletes with a strong neuromuscular baseline may be best positioned to exploit daily neurofeedback stimulation, possibly because their motor systems are already operating near an efficiency threshold where cortical modulation of frontal alpha produces downstream neuromuscular benefits more reliably. In contrast, LRG athletes, with lower baseline RMS (109.18 µV), showed the largest relative EMG amplitude increase (+17.44 µV) but inferior functional outcomes, indicative of non-specific recruitment rather than efficient motor adaptation, a pattern particularly prevalent in lower-frequency protocols that may not provide sufficient cortical priming for coordinated motor unit discharge.

These observations suggest a working framework for protocol selection: athletes with low baseline FAI and high baseline RMS appear to be optimal candidates for daily high-frequency neurofeedback; those with moderate FAI and intermediate RMS may respond adequately to every-other-day protocols; while athletes with high baseline FAI may require either longer sessions, different frequency targets, or alternative consolidation-focused designs. Future research should prospectively test this classification framework and incorporate additional baseline measures, including alpha peak frequency, theta/alpha ratio, resting cortico-muscular coherence, and psychological indices of interceptive awareness, as candidate predictors of responder phenotype. Machine-learning-based classification approaches applied to multivariate baseline profiles represent a promising avenue for individualizing neurofeedback protocols in elite sport.

### 4.6. Practical Implications

The demonstrated superiority of daily EEG biofeedback suggests that short, high-frequency interventions are preferable for eliciting robust neuromuscular and cortical gains when targeting strength, power, and fatigue resistance. For high-performance environments where training time is limited, incorporating daily neurofeedback sessions could accelerate adaptation and maximize return on neurotechnological investment. Results also support individualized periodization of neurofeedback, allowing practitioners to tailor session frequency to athlete-specific adaptation profiles. The evidence that less frequent protocols may not only be ineffective but potentially maladaptive for some neurophysiological parameters highlights the importance of evidence-based protocol selection in applied sport.

### 4.7. Implications for Wearable Biosensor Development

These findings have direct implications for next-generation wearable biosensor design for athletic performance monitoring. Key technical requirements include: (1) unobtrusive devices not interfering with movement; (2) real-time motion artifact suppression optimized for embedded processors; (3) multi-modal sensor fusion (EEG, EMG, IMU, heart rate variability); (4) biocompatible materials and sweat-resistant coatings supporting extended monitoring; and (5) secure wireless protocols compliant with data protection standards (GDPR, HIPAA). The dose–response relationships identified here provide evidence-based targets for translating laboratory protocols to field-deployable wearable platforms utilizing printed flexible sensors, energy-harvesting electronics, and edge AI inference.

### 4.8. Study Limitations

Several limitations should be acknowledged. Although an a priori G*Power analysis (d = 0.8, α = 0.05, power = 0.80) indicated that *n* = 24 (8 per group) was adequate to detect large group × time effects in the primary outcomes, this sample size may still be underpowered for smaller effects and therefore some subtle group differences or associations could have remained undetected. This elite, male, national-team cohort also limits generalizability to other performance levels, sports, and female athletes. Neurochemical markers (BDNF, fMRI) were not assessed, precluding inference regarding molecular adaptation mechanisms. Both EEG and EMG systems were laboratory-grade devices unsuitable for field deployment, and the standardized laboratory environment (22 °C, low EMI, seated posture) does not reflect dynamic athletic training conditions. Our analysis relied on offline signal processing with computationally intensive algorithms; real-time applications would require optimization for embedded architectures. No systematic comparison with gold-standard research systems (high-density EEG, intramuscular EMG) was conducted. Finally, the phenotypic stratification strategy, though theoretically grounded, may not generalize to other sports or performance levels, and individual responder effects warrant replication in more heterogeneous samples.

## 5. Conclusions

This study establishes session frequency as a critical optimization parameter for EEG biofeedback interventions in elite athletes. Daily protocols produced substantial and consistent improvements in strength, FAI, and fatigue resistance, demonstrating superior overall adaptation. The dissociation between RMS magnitude and functional outcomes suggests that adaptation quality, not simply amplitude, determines training efficacy. These findings provide robust, evidence-based recommendations for practitioners designing neurofeedback protocols in high-performance sport and establish biosensor-based methodologies with translational potential for next-generation wearable monitoring platforms. Future research should employ larger, more diverse samples, integrate multimodal biomarkers, and investigate how session frequency interacts with other protocol variables to further refine neurofeedback optimization strategies.

## Figures and Tables

**Table 1:** Technical specifications of biosensor systems.

Parameter	EEG System (actiCHamp)	EMG System (TeleMyo 2400T)
Manufacturer	Brain Products GmbH, Germany	Noraxon, Scottsdale, AZ, USA
Channels	32	16 (8 differential pairs)
Sampling Rate	1000 Hz	2000 Hz
Resolution	24-bit	16-bit
Input Impedance	>10 GΩ	>100 MΩ
CMRR	>90 dB	>100 dB
Noise Level	<1 μV RMS	<2 μV RMS
Bandpass Filter	0.1–100 Hz	20–450 Hz
Wireless Range	—	Up to 300 m

**Table 2:** Mean pre- and post-intervention values for FAI, F3, F4, STH_SUM, and RMS by protocol group.

Group	FAI_PRE	FAI_POST	F3_PRE	F3_POST	F4_PRE	F4_POST	STH_SUM _PRE	STH_SUM _POST	RMS_PRE	RMS_POST
HRG	−0.018	0.051	4.36	4.67	4.59	4.85	2.16	2.28	113.44	129.98
MRG	0.023	0.040	4.53	4.53	4.72	4.95	1.90	2.00	111.10	127.42
LRG	0.013	0.016	4.61	4.49	4.94	4.51	1.88	1.96	109.18	126.62

Note: F3 and F4 values represent log-transformed spectral power in arbitrary units (a.u.); FAI is dimensionless; RMS is in microvolts (μV).

**Table 3:** Absolute pre–post changes in key outcome measures by protocol group.

Outcome	HRG (Δ Pre–Post)	MRG (Δ Pre–Post)	LRG (Δ Pre–Post)
FAI	+0.069	+0.017	+0.003
F4	+0.26	+0.23	−0.43
STH_SUM	+0.12	+0.10	+0.08
RMS	+16.54	+16.32	+17.44

Note: FAI (Frontal Alpha Index) is dimensionless; F4 and STH_SUM are in arbitrary units derived from standardized indices; RMS is in microvolts (μV).

**Table 4:** Mean within-group correlation coefficients between RMS and external load.

Group	Mean Correlation (RMS–Load)
HRG	0.06
MRG	0.16
LRG	−0.14

**Table 5:** Changes in fatigue indices and retention-phase EMG/EEG metrics by group.

Group	MF_Drop (Δ Pre–Post)	Fatigue_Index_% (Δ)	RMS_Retention	MF_Retention
HRG	−2.15	−1.90	141.87	72.17
MRG	−1.12	−0.87	139.63	70.36
LRG	−0.24	−0.21	138.28	68.13

## Data Availability

The raw data, analytic scripts, and Supplementary Materials are publicly available in an open-access repository (Zenodo, DOI: 10.5281/zenodo.15879450, accessed on 22 February 2026), ensuring full transparency and reproducibility in accordance with best practices for high-impact experimental research. The code and detailed analysis pipeline, including R and Python scripts, are version-controlled and documented to facilitate replication and extension by future researchers. Due to ethical restrictions and participant confidentiality obligations, the complete raw dataset containing individual athlete identifiers and sensitive health information is not publicly available but is accessible upon reasonable request from the corresponding authors, subject to institutional data governance protocols and institutional review board approval.

## Supplementary Materials

The following supporting information can be downloaded at https://www.mdpi.com/article/10.3390/s26072077/s1, Supplementary S1, Table S1. Baseline demographic and neurophysiological characteristics of elite judo athletes by protocol group.

## Abbreviations

The following abbreviations are used in this manuscript:

Abbreviation	Full Form
EEG	Electroencephalography/Electroencephalographic
EMG	Electromyography/Electromyographic
FAI	Frontal Alpha Index
RMS	Root Mean Square
MVC	Maximal Voluntary Contraction
SENIAM	Surface Electromyography for the Non-Invasive Assessment of Muscles
ICA	Independent Component Analysis
FFT	Fast Fourier Transform
HRG	High-Responder Group
MRG	Medium-Responder Group
LRG	Low-Responder Group
MF	Median Frequency
Sth_SUM	Strength Sum (corrected)
EEG_SUM	EEG Summary Index
%ΔEMG	Percentage Change in EMG
SNR	Signal-to-Noise Ratio
CMRR	Common-Mode Rejection Ratio
Ag/AgCl	Silver/Silver Chloride
TTL	Transistor–Transistor Logic
SOP	Standard Operating Procedure
ICC	Intraclass Correlation Coefficient
DOI	Digital Object Identifier
ANOVA	Analysis of Variance
CI	Confidence Interval
FDR	False Discovery Rate
SD	Standard Deviation
GDPR	General Data Protection Regulation
HIPAA	Health Insurance Portability and Accountability Act
IMU	Inertial Measurement Unit
AI	Artificial Intelligence
BDNF	Brain-Derived Neurotrophic Factor
fMRI	Functional Magnetic Resonance Imaging
RF	Radio Frequency
ISM	Industrial, Scientific and Medical
Hz	Hertz
dB	Decibel
ms	Millisecond
μV	Microvolt
kΩ	Kiloohm
MΩ	Megaohm
GΩ	Gigaohm
GHz	Gigahertz
