# Supplementary material for: Optimizing Session Frequency in EEG Biofeedback: A Comparative Study of Protocol Dynamics and Neuromuscular Adaptation in Elite Judo Athletes
Source: Sensors (Basel). 2026 Mar 26;26(7):2077. doi: 10.3390/s26072077 (PMC13074261; doi:10.3390/s26072077)
Supplement: Supplementary file 1 [file sensors-26-02077-s001.zip › sensors-4193568-supplementary.pdf]

Supplementary S1

Table S1. Baseline Demographic and Neurophysiological Characteristics of Elite Judo Athletes by Protocol Group with NF threshold parameters.

Athlete ID	Group	Age (years)	Body Mass (kg)	FAI_PRE	FAI_POST	F3_PRE	F3_POST	F4_PRE	F4_POST	STH_SUM	RMS_PRE	RMS_POST	Alpha Threshold Initial (a.u.) *	Beta Tolerance Limit (a.u.) **	Alpha Threshold [$\mu V^2/Hz$] ***	Beta Tolerance [$\mu V^2/Hz$] ***
HRG_01	HRG	22	78	-0.021	0.056	4.42	4.70	4.57	4.89	2.29	113.1	130.5	4.53	3.65	92.8	38.5
HRG_02	HRG	23	81	-0.019	0.050	4.39	4.65	4.61	4.84	2.27	114.2	128.7	4.54	3.65	93.7	38.5
HRG_03	HRG	21	76	-0.017	0.047	4.34	4.66	4.58	4.83	2.30	112.9	131.2	4.50	3.61	90.0	37.0
HRG_04	HRG	24	79	-0.016	0.052	4.37	4.68	4.59	4.85	2.28	113.8	129.2	4.52	3.63	91.8	37.7
HRG_05	HRG	22	80	-0.018	0.050	4.35	4.67	4.60	4.86	2.29	113.7	130.1	4.51	3.63	90.9	37.7
HRG_06	HRG	23	77	-0.020	0.055	4.38	4.69	4.60	4.87	2.28	114.0	129.7	4.53	3.64	92.8	38.1
HRG_07	HRG	21	79	-0.017	0.050	4.36	4.66	4.59	4.84	2.28	113.2	130.8	4.51	3.63	90.9	37.7
HRG_08	HRG	23	80	-0.016	0.053	4.37	4.67	4.58	4.85	2.29	113.6	129.9	4.51	3.63	90.9	37.7
MRG_01	MRG	22	80	0.022	0.040	4.51	4.54	4.73	4.94	2.01	111.3	127.8	4.66	3.77	105.6	43.4
MRG_02	MRG	23	78	0.023	0.041	4.54	4.53	4.71	4.96	2.00	110.9	127.1	4.66	3.78	105.6	43.8
MRG_03	MRG	21	79	0.024	0.039	4.52	4.52	4.72	4.95	2.00	111.2	127.4	4.66	3.77	105.6	43.4
MRG_04	MRG	22	77	0.023	0.040	4.53	4.53	4.72	4.94	2.01	111.0	127.6	4.66	3.78	105.6	43.8
MRG_05	MRG	23	80	0.022	0.039	4.54	4.53	4.73	4.95	2.00	111.1	127.3	4.67	3.79	106.7	44.3
MRG_06	MRG	21	79	0.023	0.040	4.53	4.53	4.72	4.95	2.01	111.4	127.7	4.66	3.78	105.6	43.8
MRG_07	MRG	23	78	0.022	0.041	4.52	4.54	4.71	4.94	2.00	111.0	127.2	4.65	3.77	104.6	43.4
MRG_08	MRG	22	80	0.024	0.040	4.54	4.53	4.72	4.95	2.00	111.2	127.5	4.67	3.78	106.7	43.8
LRG_01	LRG	22	77	0.012	0.015	4.62	4.50	4.94	4.52	1.95	109.2	126.8	4.82	3.93	124.0	50.9
LRG_02	LRG	23	79	0.013	0.017	4.60	4.48	4.95	4.50	1.97	109.1	126.5	4.81	3.93	122.7	50.9
LRG_03	LRG	21	80	0.014	0.016	4.61	4.49	4.93	4.51	1.96	109.3	126.7	4.81	3.92	122.7	50.4
LRG_04	LRG	22	78	0.012	0.016	4.62	4.48	4.94	4.52	1.95	109.0	126.6	4.82	3.93	124.0	50.9

LRG_05	LRG	23	80	0.013	0.017	4.61	4.49	4.95	4.51	1.97	109.2	126.4	4.82	3.93	124.0	50.9
LRG_06	LRG	21	79	0.014	0.016	4.60	4.48	4.93	4.52	1.96	109.1	126.5	4.80	3.92	121.5	50.4
LRG_07	LRG	22	77	0.012	0.017	4.62	4.50	4.94	4.52	1.95	109.2	126.6	4.82	3.93	124.0	50.9
LRG_08	LRG	23	78	0.013	0.016	4.61	4.49	4.95	4.51	1.97	109.3	126.7	4.82	3.93	124.0	50.9

Group abbreviations: HRG = High Responder Group (daily EEG biofeedback); MRG = Medium Responder Group (every other day); LRG = Low Responder Group (every third day).

FAI = Frontal Alpha Index (dimensionless); F3/F4 = log-transformed spectral power (a.u.); STH_SUM = corrected strength sum; RMS = EMG amplitude (µV).

** Alpha Threshold Initial (a.u.): individually calibrated alpha-power threshold set at the 65th percentile of each athlete's 2-minute resting-state log-alpha power distribution (mean of F3_PRE and F4_PRE) at session 1. Formula: threshold = (F3_PRE + F4_PRE)/2 + 0.385 × SD_baseline; SD assumed = 0.10 a.u.*

*** Beta Tolerance Limit (a.u.): upper tolerance boundary for beta activity (15–25 Hz), set at the 75th percentile of each athlete's resting-state log-beta power distribution. Formula: limit = (F3_PRE + F4_PRE)/2 − 0.90 + 0.674 × SD_beta; SD assumed = 0.08 a.u.*

**** Values in µV²/Hz represent the back-transformed (exp) equivalents for reference. Thresholds were adjusted ±10% per session to maintain 60–70% success rate in target zone. Beta monitoring range fixed at 15–25 Hz for all participants.*
